# A new *Cervinotaptera* species from northern Madagascar (Hemiptera, Heteroptera, Aradidae)

**DOI:** 10.3897/zookeys.796.24540

**Published:** 2018-11-15

**Authors:** Petr Baňař, Ernst Heiss

**Affiliations:** 1 Moravian Museum, Department of Entomology, Hviezdoslavova 29a, CZ-627 00, Brno, Czech Republic Moravian Museum, Department of Entomology Brno Czech Republic; 2 Tiroler Landesmuseum, Josef-Schraffl-Strasse 2a, A-6020 Innsbruck, Austria Tiroler Landesmuseum Innsbruck Austria

**Keywords:** apterous, *
Cervinotaptera
*, Mezirinae, new species, northern Madagascar

## Abstract

A new species, *Cervinotapteratomhenryi*, **sp. n.** (Hemiptera: Heteroptera: Aradidae: Mezirinae), from Montagne d’Ambre National Park in northern Madagascar is described and illustrated. The newly described species is compared with the only other known species, *Cervinotapteraguilberti* Heiss & Marchal, 2012.

## Introduction

The first comprehensive studies on Aradidae of Madagascar and adjacent islands were provided by Ludvík Hoberlandt (1957, 1963). In the last two decades Ernst Heiss published several taxonomic papers, describing many new genera and species, and summarized all published works in a catalogue (Heiss 2012). Most recently, two new genera and five new species of aradids were published (Heiss et al. 2012, Heiss and Marchal 2012, Heiss and Baňař 2013, Baňař et al. 2016, Baňař and Heiss 2018).

The genus *Cervinotaptera* Heiss & Marchal, 2012 (Hemiptera: Heteroptera: Aradidae: Mezirinae) was erected for the apterous species *Cervinotapteraguilberti* Heiss & Marchal, 2012 from northern Madagascar (Heiss and Marchal 2012). During the expeditions of the first author in January 2015 and January 2016 to Montagne d’Ambre National Park in northern Madagascar, twelve specimens of a new species of *Cervinotaptera* were collected. The species is described in this paper.

## Materials and methods

**Figure 1.**
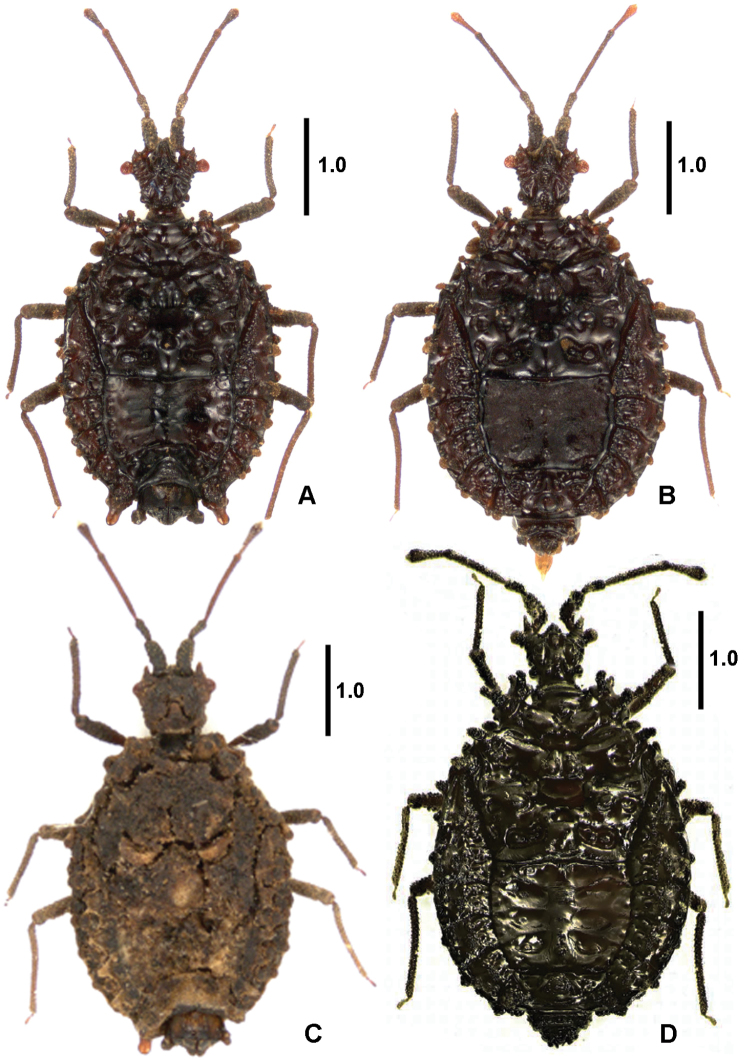
*Cervinotaptera* species, dorsal habitus. **A***C.tomhenryi* sp. n., male holotype **B***C.tomhenryi* sp. n., female paratype **C***C.tomhenryi* sp. n., male paratype, uncleaned specimen **D***C.guilberti* Heiss & Marchal, 2012, female holotype. Scale bars: 1 mm.

The body surface of most apterous, litter-living aradids is frequently covered by a layer of incrustations, which obscure body structures and intersegmental boundaries (Figure 1C). It was therefore necessary to clean the specimens before examination. In this case, we used a combination of mechanical cleaning in distilled water with common detergent and short treatment in 10% KOH.

The term "dorsal ocular index" refers to the ratio of the minimum interocular distance to the maximum width of the eye; it is best calculated if measured as: (twice minimum interocular distance) / (maximum width across eyes, minus minimum interocular distance).

Color photographs of the newly described species were taken with a Leica MSV266 camera. Scanning electron micrographs of a gold-coated specimen were taken using a JEOL 6380 LV scanning electron microscope.

Measurements were taken using a SZP 11 ZOOM stereoscopic microscope with an eyepiece micrometer. Label data are cited verbatim, including potential errors, using a slash (/) to separate lines on the label; different labels are mentioned and indicated by a double slash (//). Notes of the authors are in [square brackets].

Abbreviations used in text:

**deltg** dorsal external laterotergite (connexivum),

**mtg** mediotergite,

**vltg** ventral laterotergite,

**pe-angle** posteroexterior angle (of deltg).

The material studied is deposited in following collections:

**MMBC** Moravian Museum, Brno, Czech Republic

**CEHI** Ernst Heiss collection, Tiroler Landesmuseum, Innsbruck, Austria.

## Taxonomy

### Family Aradidae Brullé, 1836

#### Subfamily Mezirinae Oshanin, 1908

##### Genus *Cervinotaptera* Heiss & Marchal, 2012

###### Cervinotaptera tomhenryi sp. n.

Taxon classificationAnimaliaHemipteraAradidae

http://zoobank.org/0C6FF72F-8E60-47F3-B81E-3D2EF9833596

**Figure 2.**
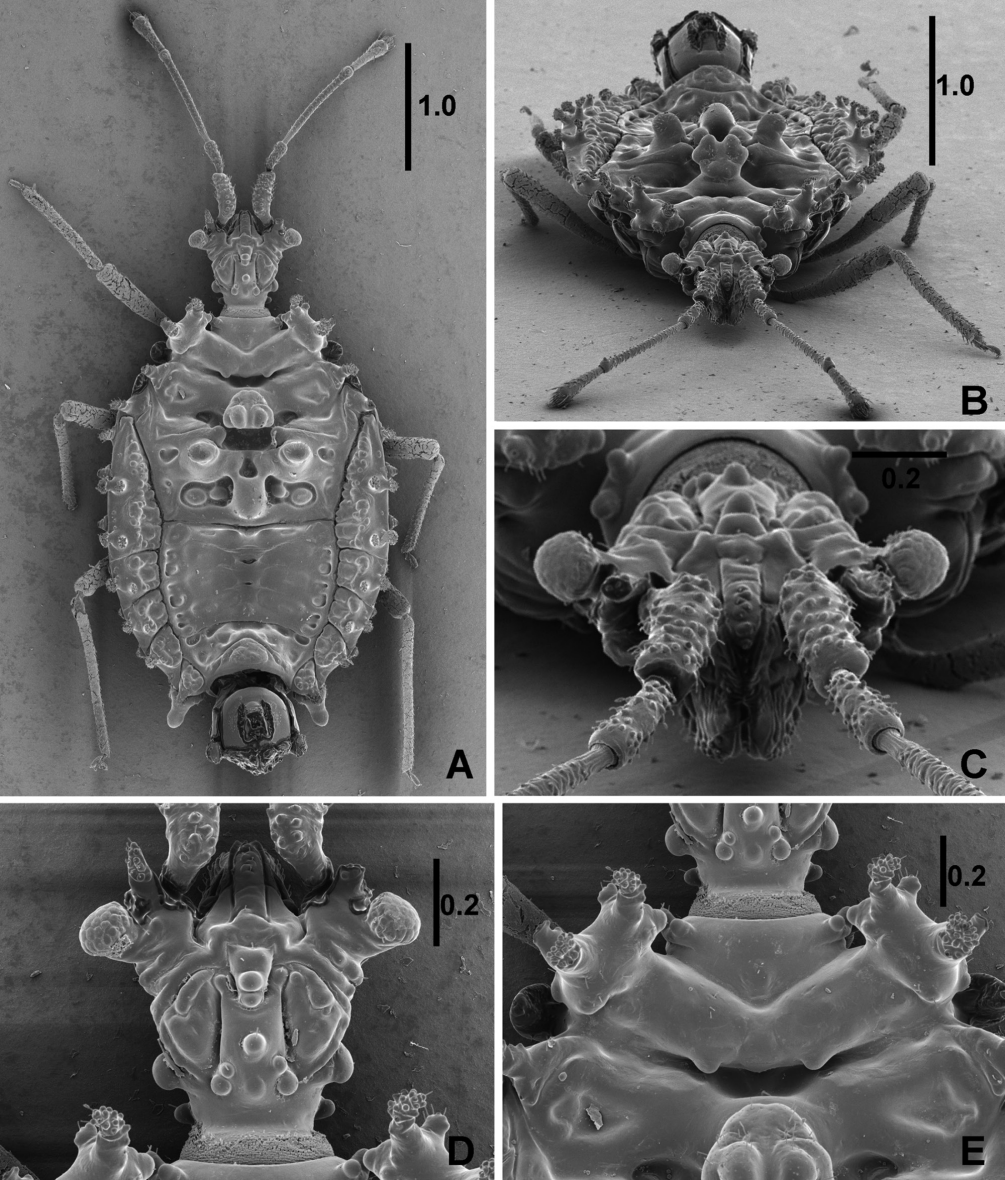
*Cervinotapteratomhenryi* sp. n., male paratype, scanning electron micrographs. **A** dorsal habitus **B** dorsal habitus, anterior view **C** head, anterior view **D** head, dorsal view **E** pronotum, dorsal view. Scale bars in mm.

**Figure 3.**
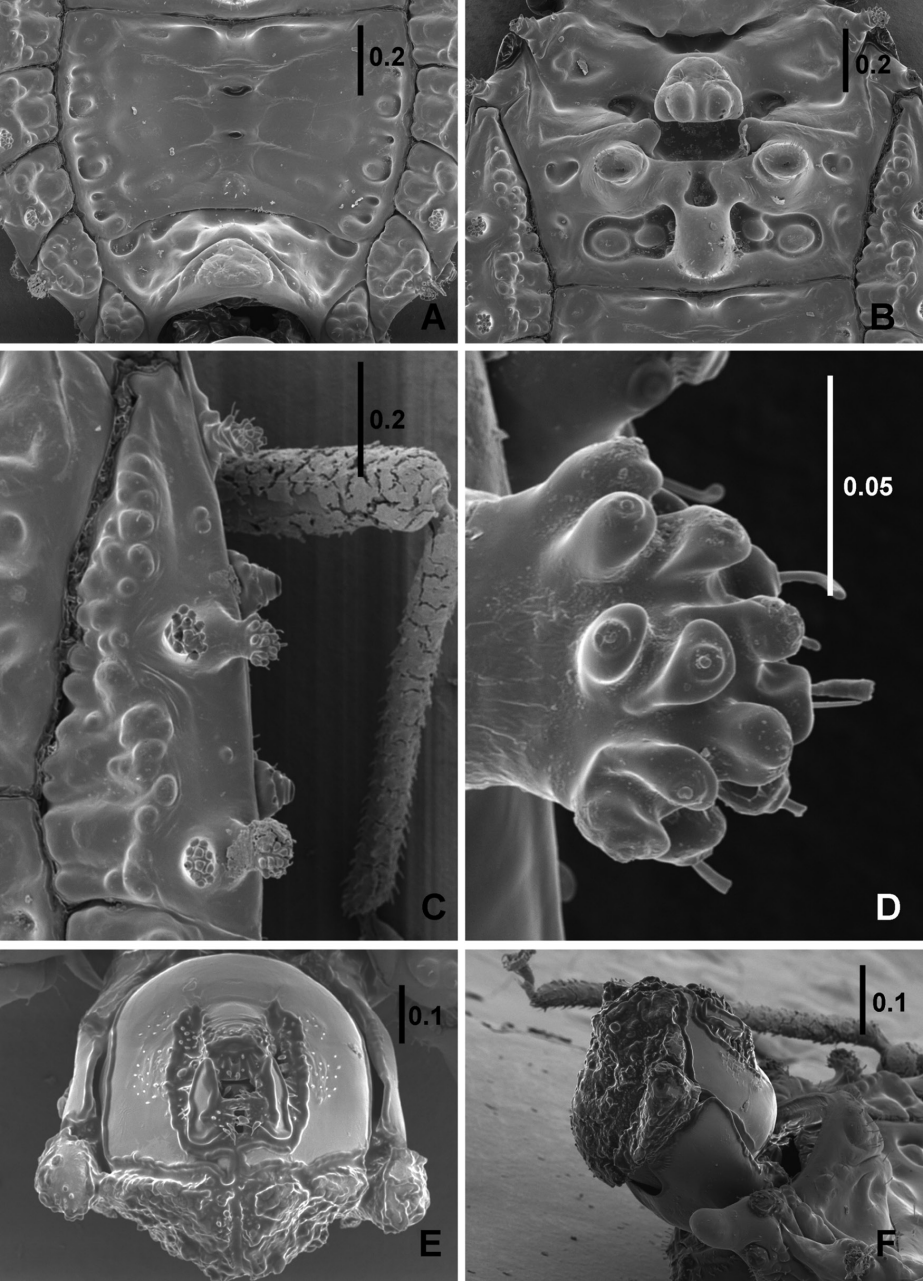
*Cervinotapteratomhenryi* sp. n., male paratype, scanning electron micrographs. **A** tergal plate **B** mesonotum and metanotum with fused mediotergites I+II **C** triangular sclerite of fused dorsal external laterotergites II+III **D** apex of tubercle of dorsal external laterotergites II+III **E** pygophore dorsal view **F** pygophore lateral view. Scale bars in mm.

**Figure 4.**
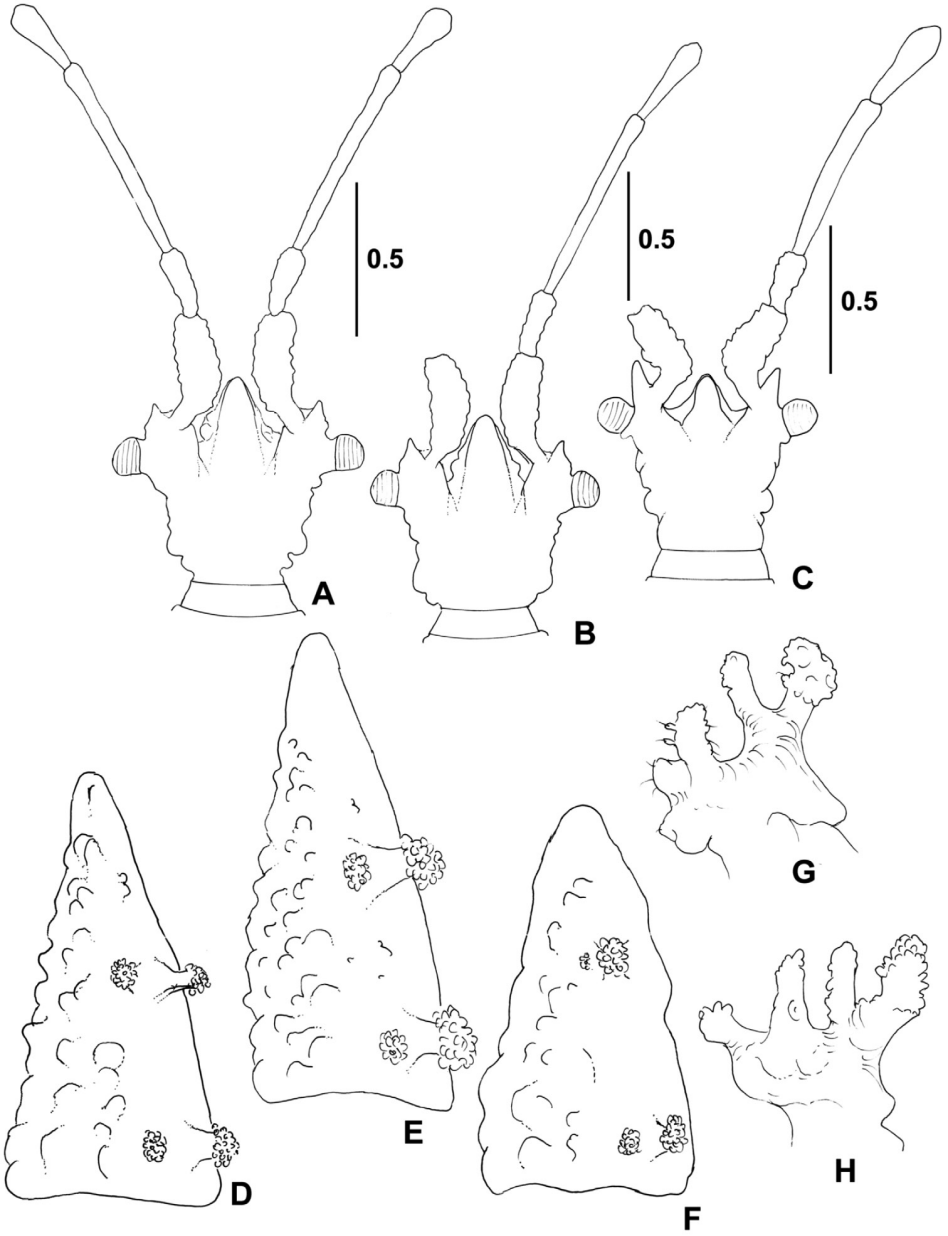
*Cervinotaptera* species. **A–C** outline of head **D–F** fused dorsal external laterotergites II+III **G–H** lateral finger-like processes of pronotum. **A, D***C.tomhenryi* sp. n., male holotype **B, E, G***C.tomhenryi* sp. n., female paratype **C, F, H***C.guilberti* Heiss & Marchal, 2012, female holotype. Scale bars in mm, **D–H** schemes, not measured.

Figures 1A–C ; 2 ; 3 ; 4A–B, D–E, G

####### Material examined.

**Type material. Holotype** male, ‘MDA/Jan.2015/11 N MADAGASCAR / MONTAGNE D‘AMBRE ~945m, circuit / „Sommet“, S12°31’28‘‘E49°09’52‘‘ / sifting litter+rotten wood, Winkler app. extr. / 14.1.2015, P. Baňař & E.M. Rabotoson lgt.’ [printed] // ‘HOLOTYPE / *Cervinotaptera* / *tomhenryi* sp. nov. / Baňař & Heiss des. 2018’ [printed red label] (MMBC). Paratypes: 2 ♂♂, 3 ♀♀, same locality label as holotype [one male gold-coated for SEM] (1 ♂, 1 ♀ MMBC; 1 ♂, 2 ♀♀ CEHI); 1 ♂, 2 ♀♀: ‘MDA/Jan.2015/12 N MADAGASCAR / MONTAGNE D‘AMBRE ~1100m / sifting litter close to camp, 16.1.2015 / Winkler apparatus extraction / P. Baňař & E.M. Rabotoson lgt.’ (1 ♀ MMBC; 1 ♂, 1 ♀ CEHI), 3 ♂♂: ‘MDA/Jan.2016/02 N MADAGASCAR / MONTAGNE D‘AMBRE 1165m, circuit / „Sommet“, S12°31’50‘‘E49°10’16‘‘ / sifting *Pandanus* litter, Winkler app. extr. / 13.1.2016, P. Baňař & E.M. Rabotoson lgt.’ [all three males permanently stored in absolute ethanol available for DNA study] (MMBC). All paratypes are provided with a label: ‘PARATYPE / *Cervinotaptera* / *tomhenryi* sp. nov. / Baňař & Heiss des. 2018’ [printed red label].

####### Description.

Apterous, body short, broadly oval (Figures 1A–C, 2A). Coloration dark brown to blackish, tarsi and apex of antennal segment IV somewhat paler. Thorax and abdominal laterotergites with tubercle-like processes.

####### Measurements

(in mm). Male holotype (one female paratype in brackets). Total body length: 3.62 (4.04); head length (without collar): 0.67 (0.71); head width across eyes: 0.84 (0.86); minimum interocular distance: 0.59 (0.60); length of antennal segments: I: 0.37 (0.39), II: 0.22 (0.23), III: 0.67 (0.69), IV: 0.37 (0.38); pronotum length [including tubercles]: 0.60 (0.60), pronotum width [including tubercles]: 1.42(1.42); maximum width of abdomen: 1.98 (2.33), tergal plate length: 0.93 (0.93); tergal plate width: 1.22 (1.33).

*Head* (Figures 2C–D, 4A–B) with longitudinal furrows and ridges and few globular tubercles on dorsal and lateral faces, numerous and more conspicuous on ventral face; wider than long, width : length ratio 1.25 in male, 1.21 in female; clypeus reaching nearly middle of antennal segment I, antenniferous lobes short, slightly shorter than clypeus; antennae long, 1.92 times as long as width of head in male, 1.95 times in female, segment I slightly bent at base, thickest, segment II thinner and shortest, segment III thinnest and longest, segment IV fusiform, antennal formula (longest segment first): III:I=IV:II. Eyes very small, globular, slightly stalked, ocular index 4.72 in male, 4.60 in female. Labium very short, hardly reaching two thirds of head length.

*Pronotum* 2.37 times as long as wide across lateral tubercles in both sexes; anterior lobe with two pairs of lateral tubercles (Figures 2E, 4G), posterior lobe smooth at middle, lateral lobes each with four finger-like processes, posterior margin convex with two small tubercles directed posteriorly. Pronotum separated from mesonotum by deep and wide furrow.

*Mesonotum* fused to metanotum, fusion lines only partly visible, posteriorly with conspicuous median elevation bearing two semicircular tubercles. Posterior margin with two deep pits connected with very deep and broad median depression on metanotum.

*Metanotum* (Figure 3B). Fused to mesonotum and mtg I+II, with rectangular median depression on anterior margin. Fused mtg I+II with deep median pit anteriorly, posteriorly with prominent median elevation, lateral parts with two (1+1) round elevations. Posterior margin of mtg I+II almost straight, clearly delimited from tergal plate by conspicuous furrow.

*Abdomen.* Tergal plate (Figure 3A) shorter than wide, ratio width to length 1.31 in male, 1.43 in female. Scent gland scars visible on posterior margins of mtg III and IV only. Deltg II+III fused to elongate triangular sclerite (Figure 3C), bearing two pairs of finger-like processes, inner process directed upwards, outer process strictly lateral. Deltg III-VII well separated from each other, bearing pair of processes similar to those on deltg II+III, shorter on deltg VI and VII. Vltg VII of male with posteriorly directed glabrous finger-like projections. Spiracles on ventral laterally produced tubercles decreasing in size from vltg II–VII, visible from above, those of paratergites VIII terminal. Metathoracic scent gland with long curved evaporatorium and additional ovate evaporatorium laterally of anterior coxae.

*Legs* unarmed, slender, sparsely covered with short, semi-erect setae. Femora widening distally, tibiae slightly curved.

*Male genitalia* (Figures 3E–F). Visible part of pygophore convex, short, and wide, surface with rugosities; parameres hook-like; paratergites VIII rounded, shorter than pygophore.

*Female.* General body structures similar in both sexes, female larger and wider.

####### Etymology.

Dedicated to our dear colleague and friend Thomas J. Henry, eminent student of the Heteroptera.

####### Collecting circumstances.

All known specimens were collected by sifting mountain evergreen rain forest leaf litter in Montagne d’Ambre National Park (Figure 6A–B) in northern Madagascar. Sifted samples were extracted in a Winkler apparatus during two or three days and were mixed several times daily. *Cervinotapteratomhenryi* sp. n. shared the microhabitat with the recently described carventine aradid *Comorocorisestherineae* Baňař & Heiss, 2018; the largest parts of the type series of both species were extracted from the same samples during the expedition of 2015.

####### Distribution.

Known only from Montagne d’Ambre National Park in northern Madagascar.

####### Differential diagnosis.

*Cervinotapteratomhenryi* sp. n. differs from *Cervinotapteraguilberti* Heiss & Marchal, 2012 by wider head; longer and thinner antennae; less curved antennal segment I; shorter antennal tubercles (Figures 4A–C); different proportions of deltg II+III sclerite (longer and narrower in *C.tomhenryi* sp. n.); larger and more prominent tubercles on deltg II+III (smaller in *C.guilberti*) (Figure 4D–F); and shape and size of finger-like processes on pronotum (Figure 4G–H).

**Figure 5.**
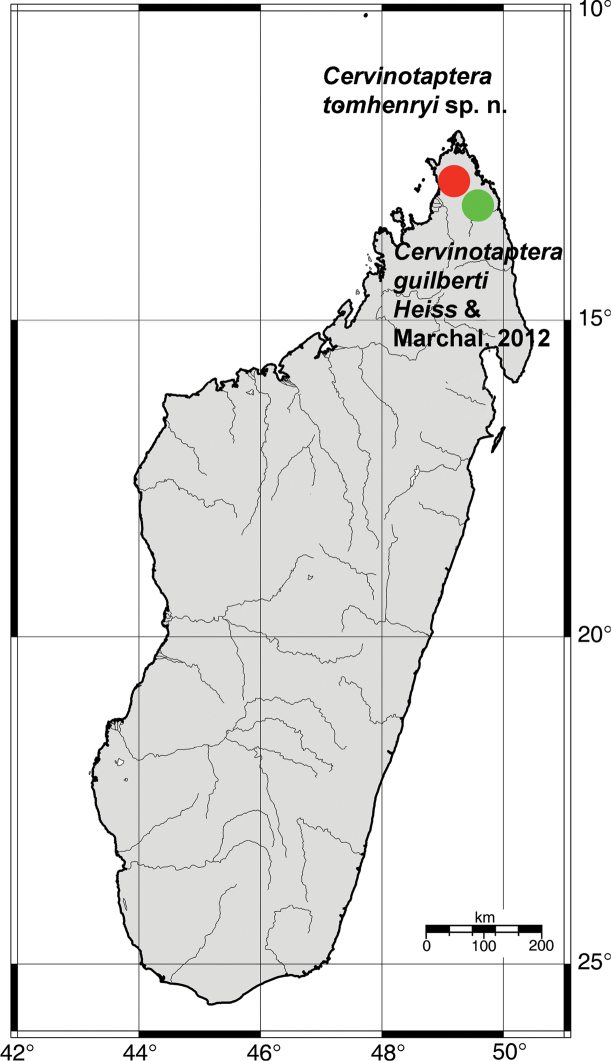
Map of distributions of *Cervinotaptera* species.

**Figure 6.**
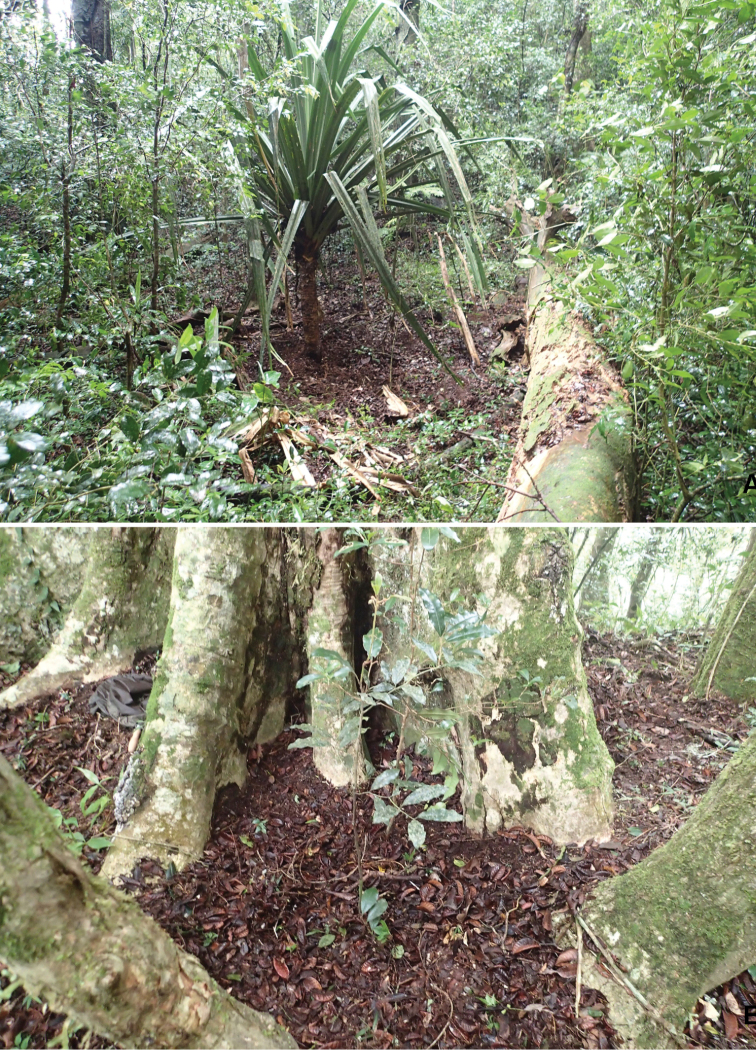
Microhabitats of *Cervinotapteratomhenryi* sp. n. in Montagne d’Ambre National Park. **A** sample MDA/Jan.2015/12 **B** sample MDA/Jan.2015/11.

## Discussion

Apterous Aradidae in stable tropical habitats of Madagascar tend to develop curious abdominal structures such as forked lateral expansions on the thorax and abdomen (*Chlonocoris* Usinger & Matsuda, 1959), ovate callosities and nodules (*Comorocoris* Heiss, 1985) or finger-like projections of different size (*Cervinotaptera*) and, in Mezirinae, show a remarkable development of unusual structures of metathoracic (*Ambohitantelya* Heiss & Baňař, 2013) and even prothoracic scent-gland evaporatoria. The adaptive value of these unusual structures and their importance in aradid classification are unknown and require further investigation.

## Supplementary Material

XML Treatment for Cervinotaptera tomhenryi

## References

[B1] Baňař P Heiss E (2018) A new species of *Comorocoris* from Northern Madagascar (Hemiptera: Heteroptera: Aradidae). Zootaxa 4375(1): 433–440. 10.11646/zootaxa.4375.3.9 29690081

[B2] Baňař P Heiss E Hubáčková L (2016) New species of *Ribesaptera* Heiss from eastern Madagascar (Hemiptera: Heteroptera: Aradidae). Zootaxa 4088(1): 146–150. 10.11646/zootaxa.4088.1.9 27394332

[B3] Heiss E (1985) Eine neue aptere Aradidengattung aus Afrika (Heteroptera, Aradidae, Carventinae). Revue Zoologique africaine 99: 147–152.

[B4] Heiss E (2012) Annotated catalogue of the flat bug family Aradidae Brullé, 1836 of Madagascar and adjacent islands (Hemiptera: Heteroptera). Zootaxa 3426: 45–63. 10.11646/zootaxa.3718.4.3 26258230

[B5] Heiss E Baňař P (2013) *Ambohitantelyayuripopovi* gen. nov. et sp. nov. a new apterous Mezirinae from Madagascar (Hemiptera: Heteroptera: Aradidae) with unique metathoracic evaporatoria. Zootaxa 3616(3): 291–297. 10.11646/zootaxa.3616.3.8 24758811

[B6] Heiss E Baňař P Rahanitriniaina LS (2012) Two new species of the apterous carventinae genus *Comorocoris* Heiss, 1985 from Madagascar (Hemiptera: Heteroptera: Aradidae). Zootaxa 3411: 63–68.

[B7] Heiss E Marchal L (2012) *Cervinotapteraguilberti* n. gen., n. sp., a conspicuous apterous Mezirinae from Madagascar (Hemiptera: Heteroptera: Aradidae). Zootaxa 3591: 84–88.

[B8] Hoberlandt L (1957) Aradoidea (Heteroptera) from Madagascar and Adjacent Islands. Acta Entomologica Musei Nationalis Pragae, Supplementum 4: 1–109.

[B9] Hoberlandt L (1963) Additional notes on Aradidae (Heteroptera) from Madagascar and Adjacent Islands. Acta Entomologica Musei Nationalis Pragae 35: 127–170.

[B10] Usinger RL Matsuda R (1959) Classification of the Aradidae. British Museum (N.H.), London, 410 pp.

